# A family of experiments about how developers perceive delayed system response time

**DOI:** 10.1007/s11219-024-09660-w

**Published:** 2024-03-04

**Authors:** Oscar Cornejo, Daniela Briola, Daniela Micucci, Davide Ginelli, Leonardo Mariani, Adrián Santos Parrilla, Natalia Juristo

**Affiliations:** 1 Interdisciplinary Centre for Security, Reliability and Trust, University of Luxembourg, Esch-sur-Alzette, Luxembourg; 2 DISCO, University of Milano - Bicocca, Milan, Italy; 3 ITEE, University of Oulu, Oulu, Finland; 4 Universidad Politécnica de Madrid, Madrid, Spain

**Keywords:** Monitoring, User studies, Empirical software engineering

## Abstract

Collecting and analyzing data about developers working on their development tasks can help improve development practices, finally increasing the productivity of teams. Indeed, monitoring and analysis tools have already been used to collect data from productivity tools. Monitoring inevitably consumes resources and, depending on their extensiveness, may significantly slow down software systems, interfering with developers’ activity. There is thus a challenging trade-off between monitoring and validating applications in their operational environment and preventing the degradation of the user experience. The lack of studies about *when* developers perceive an overhead introduced in an application makes it extremely difficult to fine-tune techniques working in the field. In this paper, we address this challenge by presenting an empirical study that quantifies how developers perceive overhead. The study consists of three replications of an experiment that involved 99 computer science students in total, followed by a small-scale experimental assessment of the key findings with 12 professional developers. Results show that non-negligible overhead can be introduced for a short period into applications without developers perceiving it and that the sequence in which complex operations are executed influences the perception of the system response time. This information can be exploited to design better monitoring techniques.

## Introduction

Modern approaches to software development consider the boundary between development-time and runtime as fading away (Baresi & Ghezzi, 2010), and several analysis solutions nowadays exploit the operational environment as a testbed to analyze and test software (Hosek & Cadar, 2015; Arnold et al., 2011; Orso, 2010; Gazzola et al., 2017; Ceccato et al., 2020).

In this context, monitoring solutions can be used to obtain data about the behaviors of developers while working on their development tasks, to support the continuous improvement of the development process (van der Aalst, 2012; Rubin et al., 2014; Couceiro et al., 2019; Meyer, Barton et al., 2017; Meyer, Murphy et al., 2017; Züger et al., 2017). Indeed, the collected data can be used to improve many aspects, such as development practices (Meyer, Barton et al., 2017), software tools (Meyer, Murphy et al., 2017), and collaboration between developers (van der Aalst, 2012).

Running monitoring and analysis activities in parallel with development activities may annoy developers. Since the available resources are shared between all the processes running in a same environment, the analysis processes may introduce slowdowns in the user processes, negatively affecting the user experience of the developers. This is particularly true for *desktop* *interactive applications*, that is, applications that continuously interact with users (differently from background applications, to make an example), such as the integrated development environments (IDEs) typically used by developers to implement their software. Unfortunately, *little is known about the actual user perception of any slowdown* introduced in interactive applications, and thus, embedding analysis strategies in software programs without annoying the developers can be challenging.

There are studies about the absolute perception of time (Seow, 2008; Killeen & Weiss, 1987), about the capability to perceive small deviations in durations (Killeen & Weiss, 1987), and studies investigating the impact of slowdowns in specific situations, especially while browsing the Web (Nah, 2004; Hoxmeier & Cesare, 2000). However, the capability to tolerate and detect slowdowns in desktop interactive applications has never been studied systematically, *neither in the context of software development nor in broader contexts*. Identifying to what extent developers can recognize slowdowns is extremely important because it can be used to quantify the amount of resources that can be consumed by any additional analysis process embedded in a program running in the field, including development and productivity tools.

This paper extends the initial evidences reported in Cornejo et al. (2017a, 2020a) with an extensive study based on human subjects about the impact of monitoring on system response time. In particular, this paper presents an empirical study that quantifies if and to what extent the overhead introduced in an interactive application is perceived by users, considering the specific case of developers interacting with their IDE (please note that in the remaining of the paper, we use the terms *user* and *developer* in an interchangeable way, since the users of the considered applications are developers), to finally discover how much the monitored software can be unnoticeably slowed down.

This is a key contribution that can perspectively allow the definition of more efficient, in particular less detrimental for the system response time, monitoring solutions, as preliminary investigated later on in this paper too.

The study consists of three replications of the same experiment (that is, a *family of experiments*), involving a total of 99 computer science students who provided information on their perception of the system response time while using Eclipse. Since a user might execute different kinds of operations of different complexity and different durations while the system is slowed down, ranging from opening a menu to compiling a program, we expect that the perception of the overhead may depend on the nature of the operation that is executed and on its context. For this reason, we consider multiple *categories of operations* and sequences of operations executed in different *order*, investigating whether these variables influence one another.

Our study produces findings that can be exploited to carefully design monitoring and analysis procedures running in the field, within development tools (e.g., IDEs). In particular, we observed that (1) developers are unlikely to recognize significant overhead levels, up to 80%, if introduced for a small number of interactions (about 4 in our experiment); (2) developers might be slightly more sensitive to overhead introduced in operations that require a significant processing time (between 5 and 10 s in our experiment); and (3) the context of execution of an operation (e.g., the operations recently executed) may impact the perception of the overhead. We assessed these findings with a small-scale experiment involving 12 professional developers. The results of this study confirm the initial findings while suggesting a stronger dependence on the context of execution of the operations for professionals compared to students.

Our study focuses on developers who use IDEs, but it represents an instance of a more general case, that is, the case of users who interact with applications while having an expectation on the system response time based on experience. Our findings might also be useful to fine-tune monitoring techniques when the monitored applications are used by knowledged users.

The presentation of the study is organized as follows. Section 2 describes the operations that can be performed by users and their categorization from the point of view of the expected system response time. Section 3 presents the setting of our experiment, including the goal and the research questions. Sections 4 and 5 describe the design alternatives and the final design that we adopted for our family of experiments. Section 6 presents the analysis of the results. Section 7 summarizes the main findings of our study, which are assessed in a small-scale experiment involving 12 professional developers described in Section 8. Section 9 discusses threats to validity. Section 10 illustrates how the reported results can be translated into actionable findings. Finally, Sections 11 and 12 discuss related work and provide final remarks, respectively. We also shared online at https://github.com/danielabriolaUnimib/SRToverheadExperiment all the collected data and the documents used in the experiments.

## Operation categories

How the overhead introduced in an application is perceived is likely dependent on the kind of operation that is affected by the overhead. For instance, users may perceive the delay on the opening of a menu compared to the delay on the processing time of a longer operation differently. For this reason, we explicitly considered the type of operation as a factor in the study.

To classify operations, we relied on existing studies from the human-computer interaction community. In particular, there are some interesting studies (Seow, 2008; Shneiderman, 1987; Shneiderman et al., 2009) that correlate the nature of the operation to its expected system response time or *SRT* for short, that is, the time elapsed between the user request and the response of the application. For the purpose of our study, we used the categorization defined by Seow (2008) because he designed his study considering the interaction between the users and the computer as a conversation, that is consistent with the behavior of interactive software applications like the ones we considered.

We thus used the following categories:

- *Instantaneous*: These are the most simple operations that can be performed on an application, such as entering inputs or navigating throughout menus (SRT, 200 ms at most).
- *Immediate*: These are operations that are expected to generate acknowledgments or very simple outputs (SRT, 1 s at most).
- *Continuous*: These are operations that are requested to produce results within a short time frame to not interrupt the dialog with the user (SRT, 5 s at most).
- *Captive*: These are operations requiring some relevant processing for which users will wait for results, but will also give up if a response is not produced within a certain time (SRT, 10 s at most).

Some operations may last longer than 10 s. However, long computations, such as operations requiring 15 s or more, are not normally perceived as part of a dialog by users (Nielsen, 1999; Miller, 1968); we thus do not consider them.

It is important to remark that operations belonging to these categories are not executed in random order. In fact, interactions with interactive applications typically consist of a sequence of short and *simple* operations (e.g., operations on menu items to reach a given window and interactions with input fields to enter data) followed by the execution of a *complex* operation (e.g., an operation that executes significant business logic). This interaction pattern is repeated many times while using an application. If we map this to Seow’s classification, we obtain interactions composed of a sequence of instantaneous and immediate operations, followed by either a continuous or a captive operation. We consequently consider this pattern when studying how the order of execution of the operations may influence the perception of the SRT.

## Experiment setting

This section provides detailed information about the experiment goals, experimental subjects, and research questions, according to Juristo and Moreno (2013). The next sections discuss the specific design that we adopted.

### Goal and research questions

The goal of this experiment is to evaluate if and when developers perceive delays in the SRT for different operation categories, as discussed in Section 2. The study is conducted under the perspective of software developers and designers interested in investigating how much overhead can be introduced in the applications without modifying the perception of the response time. This quantification is useful to design appropriate monitoring, analysis, and testing procedures running in the field within development tools (but more in general, might be of interest for anyone who needs to design tools that may affect the SRT of interactive applications).

Our study is organized into three research questions:

**RQ1:** *To what extent do different overhead levels impact developers’ perception?*

This research question investigates if and when developers recognize the overhead in a software application.

**RQ2:** *Is the perception of the overhead dependent on the operation category?*

This research question investigates whether, and when, the perception of the response time depends on the type of operation categories defined in Section 2.

**RQ3:** *Does the order of execution of complex operations affect the developer’s perception of the response time?*

This research question investigates whether the perception of the overhead introduced in complex operations may depend on their order of execution.

Since we specifically study how delays are perceived by software developers, and thus developers are the users of the application considered in our study, we interchangeably use the terms users and developers in the rest of the paper.

### Hypotheses, factors, and treatment levels

We study as factors of our experiment how developers perceive the response time when exposed to different *levels of overhead*, considering multiple *types of operations* executed in *different orders*.

Since reasoning about the human perception of the durations of phenomena requires relative measures, as supported by Weber’s law (Killeen & Weiss, 1987; Seow, 2008), we study the relative time overhead introduced in a certain operation as one of the factors to measure the perception of the response time. We define *overhead* as the computation time in excess an operation takes to complete, in comparison to its original computation time, when delay is introduced.

As treatment levels for the overhead level factor, we selected the values from the results we obtained in Cornejo et al. (2017a, 2020a), where we investigated how progressively slowing down a system may affect the resulting system response time. These previous studies identify $20\%$, $80\%$, and $140\%$ as interesting overhead values based on in-vitro experiments and the analysis of specific scenarios (e.g., tracing function calls). Here, we study how developers react to overhead introduced in actual operations, exploiting these preliminary results to design the experiment. We include the overhead value $0\%$ in the study to have a baseline to compare to.

In addition to considering the impact of relative overhead values on the perceived system response time, we also consider the *total SRT* of the operations in our analysis. That is, since the overhead increases the response time of an operation to a value which might exceed the expected SRT (*slow operation*) or not (*in time operation*), the analysis of the total SRT offers an additional perspective on the experience of the subjects and helps us deriving sound conclusions.

To cover different types of operations, we selected the four operation categories described in Section 2 as treatment levels for the operation category factor: instantaneous, immediate, continuous, and captive. As described later in Section 4, we need the subjects of the experiment to perform a sequence of operations, a *task* (see Section 5.2), on a real application, that is, we have to specify a sequence of concrete operations, each one belonging to a specific operation category. Since usually the complex operation in a task implements the functionality that the user intends to finally perform in an interaction sequence, we investigated how task order may affect the perception of complex operations. In particular, we considered the perception of the response time when a task ending with a continuous operation (long operation, requiring till 5 s to produce a result) is followed by a task ending with a captive operation (longer operation, requiring from 5 to 10 s to produce a result), and vice versa. These two orders are the treatment levels for the operation categories order factor.

More details regarding the rationale and the adopted values for the experiment are reported in Section 4.

We use the following null and alternative hypotheses for our research questions:

The null hypothesis tested to address RQ1 is as follows: $H_{0}$: *different overhead levels do not impact developers’ perception of the system response time*. The alternative hypothesis is as follows: $H_{1}$: *different overhead levels do impact developers’ perception of the system response time*.

To answer RQ2, we analyze if the category of an operation relates to the resulting perception of the response time when the operation is exposed to overhead. The null hypothesis tested to address RQ2 is as follows: $H_{0}$: *The perception of the SRT does not depend on the operation category when the operation is exposed to overhead.* The alternative hypothesis is as follows: $H_{1}$: *The perception of the SRT does depend of the operation category when the operation is exposed to overhead.*

To answer RQ3, we considered how the order of execution of complex operations may affect the perception of the overhead. For instance, running a long operation (a captive operation) followed by a slightly shorter operation (a continuous operation) may make the perception of the SRT of these operations different from the perception generated by the opposite order. The null hypothesis tested to address RQ3 is as follows: $H_{0}$: *The order of execution of tasks ending with continuous or captive operations does not influence the developer’s perception of the SRT of these operations.* The alternative hypothesis is as follows: $H_{1}$: *The order of execution of tasks ending with continuous or captive operations influences the developer’s perception of the SRT of these operations.*

In Sections 4 and 5, we describe how we limited the number of possible factors and treatment combinations to get a manageable configuration.

### Experimental subjects

For all the replications, our subjects were first-year bachelor students from the CS department of the University of Milano–Bicocca who have previously taken the same Java programming course, although not all students took the exact same edition of the course.

We recruited a total of 99 computer science students who all completed a demographic questionnaire before of the actual experiment (reported online at https://github.com/danielabriolaUnimib/SRToverheadExperiment). All students participated on a voluntary basis, and no specific incentive was in place.

Almost the entire population of students have used Eclipse. In fact, 99% of the students used Eclipse, and 35% of them exactly the same version used in the experiments, and they are all already familiar with the operations performed in the tasks of the experiment. The participants are mostly males (93 males and 6 females) with an average age of 21 years. According to these characteristics, the subjects can be considered to be representative of a population of young developers of software applications.

### Response variables and metrics

All research questions require a response variable that can measure the perception of response time (Juristo & Moreno, 2013): since we are studying how users “perceive the System Response Time,” we choose the *perceived response time* (*PRT*) as our response variable, which has already been widely used in empirical studies as a quality measure of usability (Song et al., 2014; Tu et al., 2001). This response variable can be interpreted to answer all the RQs.

We measure *PRT* using a Likert scale, where participants can express their perception with respect to the system response time. This scale goes from “Too slow” to “Too fast” divided into five levels, with the third level corresponding to “Normal” (even if it may appear unlikely that a user perceives an application as running “Too fast,” this value has to be included in the responses to have a balanced set of options that do not suggest to subjects that we were slowing down the application under test).

The response variable is measured after executing a task (a sequence of operations) with the object program, so that the users can actually express their opinion about the system they just interacted with.

To replicate the case of a user who interacts with a *known application* with a *known SRT* affected by overhead, we selected Eclipse, since our subjects have been using this IDE regularly during their programming classes in the same laboratory where the study took place.

## Design decisions

In this section, we discuss the design of the experiment, made of three exact replications (needed to involve the 99 participants), all performed in the same laboratory over 2 years, involving students after they just completed the same Java Programming course (with the same exercises and teachers).

The *factors* we are studying are as follows:

- Overhead level
- Operation category
- Operation categories order

Note that we need concrete tasks to be executed by the subjects: nevertheless, concrete tasks are not factors in this study, and we thus selected four tasks comparable in the sequence of operations that are executed (considering the operation categories in Section 5.2), duration, and complexity, and we made each participant perform all of them only once so that to avoid the learning effect during the experiment. In the remaining of this section, we discuss the possible designs of the experiment and our decisions to control the factors and their combinations, to reach the final design we adopted for the experiment (fully described in Section 5).

### Design decision 1: two operation categories orders studied

We consider Seow’s four SRT categories as operation categories, so tasks are composed of operations each one belonging to one out of four possible categories. For the purpose of the design, a task can be represented as a sequence of operation categories. For example, a task can be represented with the sequence “Instantaneous, Captive, Immediate, Continuous,” while another task could be represented with the sequence “Instantaneous, Immediate, Captive.” If we assume to have tasks with four operations, covering every possible combination of SRT operation categories requires covering 128 cases, which is not feasible.

As preliminary discussed in Section 2, not all the operation categories orders are relevant in practice. Users typically interact with applications by executing an initial sequence of simple operations, such as clicking on menu items and browsing between windows, to reach an operation of interest, which is typically a complex operation. So, to resemble reality, we should consider tasks composed of simple operations at the beginning and a complex operation at the end. This is also consistent with our practical experience on the definition of tasks in Eclipse (the selected application for the experiment) where it was almost impossible to reach a complex operation (a continuous or captive operation) without first executing a sequence of simple operations (instantaneous or immediate ones) from any state of the system. Note that the shortcut commands in Eclipse are fast operations, and they usually open windows where the user can perform the desired (complex) operation. Online at https://github.com/danielabriolaUnimib/SRToverheadExperiment, we report the concrete tasks executed by the subjects.

These considerations led us to define *two main sequences of operation categories* to be used for our study, concretized into two main types of tasks: a task with some simple operations at the beginning (both instantaneous and immediate) and a continuous operation at the end, and another task also with simple operations at the beginning but a captive operation at the end.

### Design decision 2: multiple concrete tasks

The two types of tasks that we identified can be mapped into many concrete tasks by choosing different concrete operations of the right operation category each time. To keep the study small, while still considering more than one concrete operation for each operation category, we consider *two concrete tasks for each categories order chosen*, obtaining four concrete tasks in total (detailed in Section 5.2 and shared online at https://github.com/danielabriolaUnimib/SRToverheadExperiment).

### Design decision 3: overhead orders not studied

We selected four overhead levels to be studied (as discussed in Section 3.2).

If we consider that every operation in a task can be exposed to a different overhead level and we consider every combination, we obtain 256 combinations of overhead levels to be investigated.

We decided to block the overhead orders, so instead of applying a different overhead to each operation in the task, we apply the same overhead to all the operations in each task. In this way, we reduce the cases to be considered to four: one for each overhead value we use in the experiment.

This overhead order limitation is reasonable. If we consider that tasks are made of four operations, with a total duration of less than 10 s, it makes little sense to continuously change the overhead level, while it makes sense to change the overhead level in a different task.

### Possible setups

Based on these design decisions and using the factors of Table 1, we identified two main options for the setup of the study, discussed below.

#### Between overhead levels, within operation categories, and operation categories orders

In this setup, every group of participants performs all the tasks (and consequently executes operations belonging to all the categories) while being exposed to the same overhead level.

In this way, we eliminate any issue about exposing the same participant to multiple overhead levels (it is a between overhead level setup). Since we expose each group to the same overhead level, we need enough participants in each group to be sure that the results do not depend on the group.

This design addresses the “operation categories order” factor by having half of the groups performing the tasks ending with a captive operation first and the other half executing the tasks ending with continuous operation first.

#### Within overhead levels and operation categories, between operation categories order and overhead levels

In this alternative setup, each group of participants performs all the tasks (and consequently all the operation categories) in one of the two orders, while each task is exposed to a different overhead. Since we study four overhead levels and we have four tasks, we have 64 possible combinations. This number of combinations requires the involvement of a huge number of participants in the experiment, which makes the design infeasible in practice.

We thus finally decided to adopt the first setup, which is presented in greater details in the next section.

## Experiment design

This section describes the design of the experiment that we adopted for each of the three replications.

### Experimental groups

Based on the objective of the study and the design decisions discussed in Section 4, we have clear constraints on the experiment. In particular, the study has to cover the following items:

1. Four overhead levels
2. Four operation categories
3. Two operation categories orders

Table 1 summarizes the chosen factors and treatment levels that we considered in our experiment, while the design is shown in Table 2.

**Table 1:** Factors and treatment levels

**Factors**	**Treatment levels**
Overhead level	0%, 20%, 80%, 140%
Operation category	Instantaneous, immediate, continuous, captive
Operation categories order	Continuous-captive, captive-continuous

**Table 2:** Design

**Tasks**	**Operation category order**	**Applied overhead level**			
		**0%**	**20%**	**80%**	**140%**
T3 T4 T1 T2	Continuous-captive	G1	G3	G5	G7
T1 T2 T3 T4	Captive-continuous	G2	G4	G6	G8

During each replication of the experiment, all the subjects have been homogeneously distributed in eight groups G1–G8 (to finally have in the overall at least 12 subjects for each group): groups G1, G3, G5, and G7 first perform the two tasks terminating with a continuous operation and then the two tasks terminating with a captive operation, while groups G2, G4, G6, and G8 do the opposite. This allows to study the impact of complex operations order on the perception of the overhead. In addition to continuous and captive operations, the tasks include enough instantaneous and immediate operations (see description of the tasks in next subsection) to be able to study the operation category factor.

Each group works with the same overhead level for all the performed tasks.

Since the design is *between overhead levels*, that is, each group performs all the tasks with the same overhead, we might confound the effect of the overhead with the effect of the group. To mitigate this threat, we assigned people to groups randomly, avoiding any bias in the definition of the groups.

### Tasks

The experimental objects are the tasks that the subjects perform during the experiment, reflecting some of the different studied treatments  (Juristo & Moreno, 2013). In our case, the experimental objects are the four concrete tasks that the participants have to perform on Eclipse. We included in the Eclipse workspace the JXSEConsolidation Java project that implements a P2P sharing platform. The project consists of 12 packages and 650 Java classes.

The students regularly use Eclipse for their programming tasks, so they know the IDE well. We gave the students access to the documentation of the project and gave them time to look at the structure of the code and understand the size of the project (the knowledge of the implementation is not needed to understand the tasks). Students did not resolve any compilation or build problem, since the project was pre-loaded in the Eclipse environment.

To design these four tasks, we first identified the operations implemented in Eclipse that are either continuous or captive according to Seow’s classification, and we also identified the shortest sequence of operations that must be performed to reach the identified operation and run it. Among these tasks, we finally selected the cases with the most balanced presence of operations of the other categories (instantaneous and immediate). In the ideal case, we would like to have one instantaneous and one immediate operation in each task. However, this was not always possible, and we had to tolerate the presence of two instantaneous operations in two tasks.

The resulting four tasks are as follows:

- *Task1 - Clean &Build*: Click on *Project* menu and wait for the sub-menu being visualized (instantaneous), click on *Clean* and open the dialog window (immediate), and click on *Ok* to start the *Clean &Build* operation, and wait for the progress window to be automatically closed (captive).
- *Task2 - Search*: Open the search window by clicking on the keyboard *Ctrl + H* (immediate), click on *Java Search* tab (instantaneous), enter the string C* (instantaneous), and click on *Ok* to start the search and wait for the automatic closing of the dialog window (captive).
- *Task3 - Type Hierarchy*: enlarge the project tree with a double click (instantaneous), select the *src* package with one click (Immediate), and press *F4* to start the creation of the Type Hierarchy (continuous).
- *Task4: Sort Members*: Click on the arrow near the name of the project to expand its structure (instantaneous), select the *working* package with a click (immediate), click on the *Source* menu (instantaneous), and click on *Sort Members* and wait for the end of the sorting operation (continuous).

The total set of operations executed by performing the four tasks are six instantaneous operations, four immediate operations, two continuous operations, and two captive operations.

The operation category order factor is exercised by running the tasks in two different ways: (1) to achieve the continuous-captive order, participants execute tasks in the order Task3 - Task4 - Task1 - Task2, and (2) to cover the captive-continuous order, participants execute tasks in the order Task1 - Task2 - Task3 - Task4.

To make sure the entire procedure and the tasks are clear, we had a pilot study with colleagues, Ph.D. students, and students who directly collaborated with the authors of this paper, who accepted to help us with the design of the experiment. We had multiple sessions, collecting feedback useful to improve the material at each session. We experienced no issues with the design of the experiment and the chosen tasks, and we mainly received feedback that helped us improve the experimental material, and the descriptions and questionnaires shared with the students.

### Experiment procedure

**Fig. 1:**
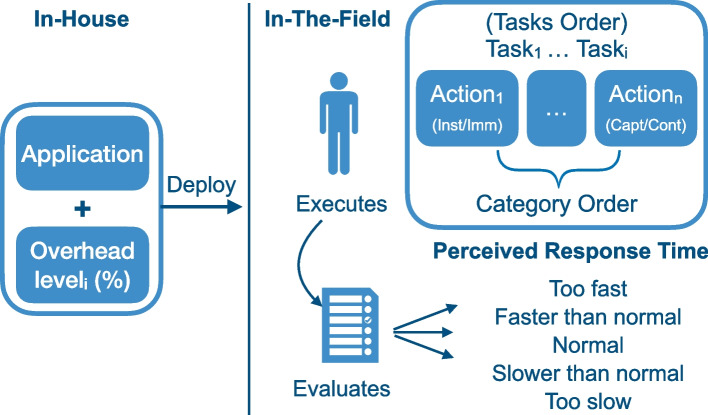
Experiment overview

The experimental procedure resembles the general structure represented in Fig. 1 and the detailed design shown in Table 2. In particular, all the participants to the study have been invited to join an experimental session in the lab they use for their courses, which hosts 100 HP ProDesk 600 G1 SFF equipped with an Intel i3-4130 and 4GB of RAM. We invited students who have taken part to Java Programming courses in our department: participation was purely based on volunteers, and no specific incentive was promised.

To have a balanced number of people in each group, we asked the students to subscribe for the experimental session in advance. We then randomly distributed the students among the eight groups.

The lab session started with a profiling questionnaire, then we distributed an instruction sheet giving general information about the structure of the experiment and a general description of the tasks that will be performed with Eclipse. Subjects were not told about the real aim of the experiment (to avoid introducing any bias in the evaluation), nor about the fact that tasks had overheads; they were simply told that we were studying the system response time of some functionalities in Eclipse.

Each participant performed the four tasks presented in Section 5.2 but in a different order (tasks 3, 4, 1, 2 for the odd groups and tasks 1, 2, 3, 4 for the even groups) and exposed to a different overhead level according to the design shown in Table 2.

Each participant incrementally accessed the four sheets describing the specific tasks to be performed with Eclipse, that is, the sheet with the description of the next task was accessible only once the previous task was completed. Each task is carefully described with text and screenshots to avoid misunderstandings. Once a task is completed and before moving to the next task, the participant evaluated the perceived response time of each operation in the task according to five possible levels: “Too slow,” “Slower than Normal,” “Normal,” “Faster than Normal,” “Too Fast.”

The subjects finally compiled an exit questionnaire to check whether the performed operations were clearly explained.

We report online at https://github.com/danielabriolaUnimib/SRToverheadExperiment all the documents we gave to the participants.

To expose each instance of Eclipse to the right overhead, we used AspectJ and Equinox Weaving (Eclipse Community, 2019) to monitor the original SRT of the Eclipse functionalities and to modify the SRT of each operation under analysis based on the group of the participant. In particular, we computed the average SRT time requested by each operation from five automatic executions of the tasks, obtained by repeating the execution of a SikuliX^1^ script in the same lab used for the experiments with the participants. We introduce a specific overhead for each user by adding a delay that depends on the group of the user. We report data about the resulting runtime and its variance in Section 6.1.

## Family analysis

This section provides descriptive data about the SRT of the operations in the tasks, when affected by the various overhead levels, presents the techniques used to study the experimental data, and finally describes the achieved results. The next section interprets the results distilling the key findings of the paper.

### Descriptive data about the SRT of the operations in the tasks

**Fig. 2:**
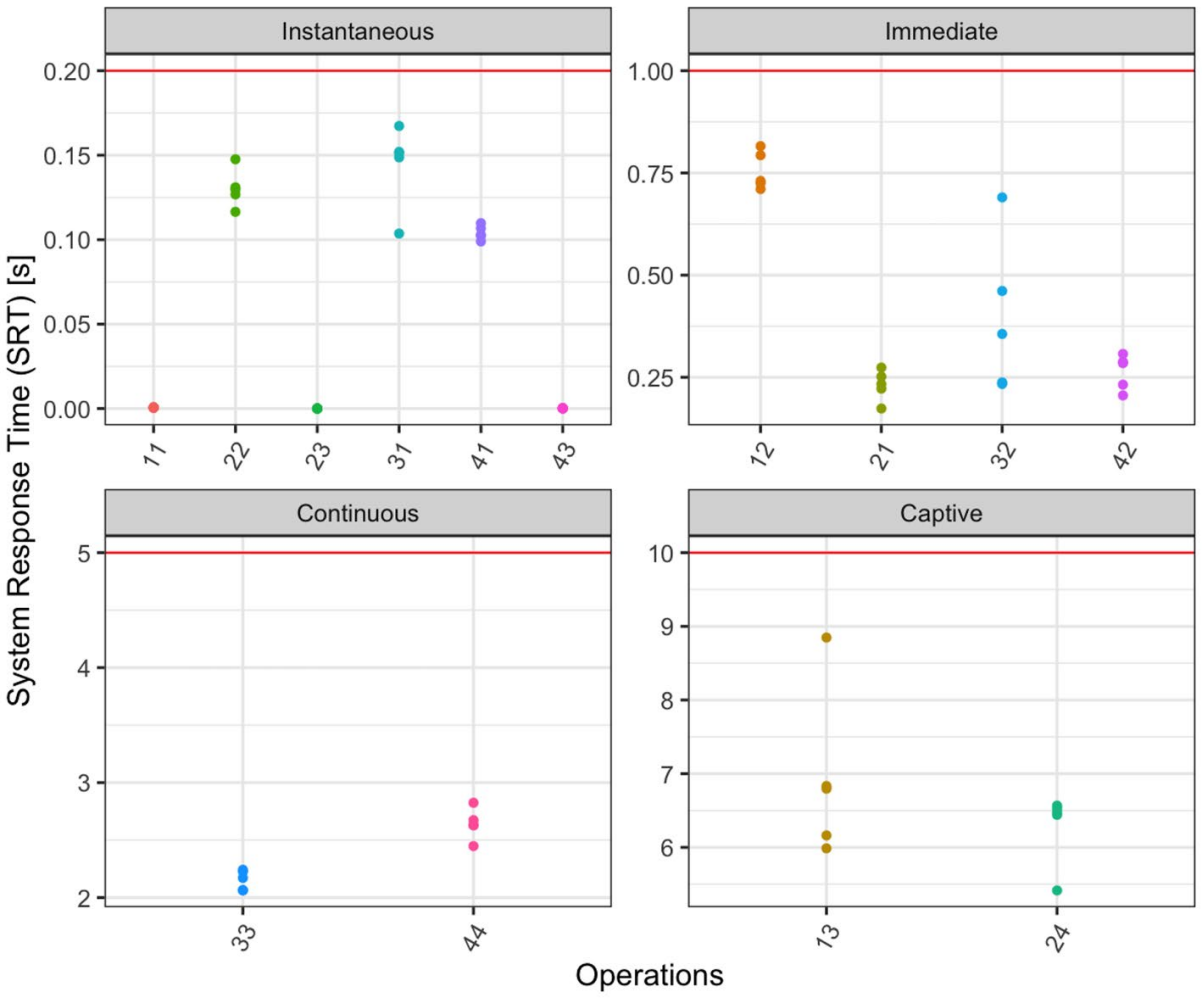
SRT values for all the considered operations. The horizontal red line shows the maximum expected time for the operations in that category

The category assigned to each operation in each task depends on its mean SRT computed from five executions of the tasks. We show the distribution of the collected samples in Fig. 2. Table 3 reports the variance, median, mean, and relative standard deviation of the execution time collected of each operation. Note that we named each operation with a two-digit number *TO*, where *T* is the number of the task and *O* is the number of the operation inside the task (e.g., operation 23 is the third operation of the second task).

All the values are below the boundary of their category. The noise in the measurements, measured as the relative standard deviation, is also low. In fact, it is always below 16%, with the exception of two operations. The relative variance is high for operation 43, but this is caused by the extremely small values of the execution time of the operation. Indeed, it is a click on a menu with an almost 0 ms execution time and with a variance that approximates to 0. Thus, its behavior is pretty consistent and it is not likely to represent a problem for the study. The immediate operation 32 is the only operation showing a relatively significant variability in the measurement. We take this into account in the discussion of the results.

**Table 3:** Absolute times (seconds) of executed tasks

**Name**	**Operation**	**Cat**	**Variance**	**Median**	**Mean**	**Relative Std Dev**
Click on Project menu	11	Inst.	0.0000000	0.0006000	0.0006000	11.79%
Click on Clean	12	Imm	0.0021601	0.7305446	0.7549467	6.16%
Click on Ok	13	Cap	1.2957357	6.7954000	6.9248000	16.44%
Ctrl + H	21	Imm	0.0014068	0.2337275	0.2310553	16.23%
Click on Java search tab	22	Inst	0.0001257	0.1297000	0.1303200	8.6%
Enter C* string	23	Inst	0.0000000	0.0000300	0.0000300	0%
Click on Ok for search	24	Cap	0.2390960	6.4777000	6.2862060	7.78%
2click on Project tree	31	Inst	0.0005775	0.1511000	0.1445400	16.63%
Select SRC package	32	Imm	0.0359490	0.3560412	0.3957981	47.90%
Press F4	33	Cont	0.0074012	2.1710000	2.1535800	3.99%
Click on name project	41	Inst	0.0000175	0.1026900	0.1041167	4.02%
Select working package	42	Imm	0.0018230	0.2848290	0.2636078	16.20%
Click on Source menu	43	Inst	0.0000000	0.0001000	0.0001400	39.12%
Click on Sort Members	44	Cont	0.0180414	2.6316000	2.6412524	5.09%

**Fig. 3:**
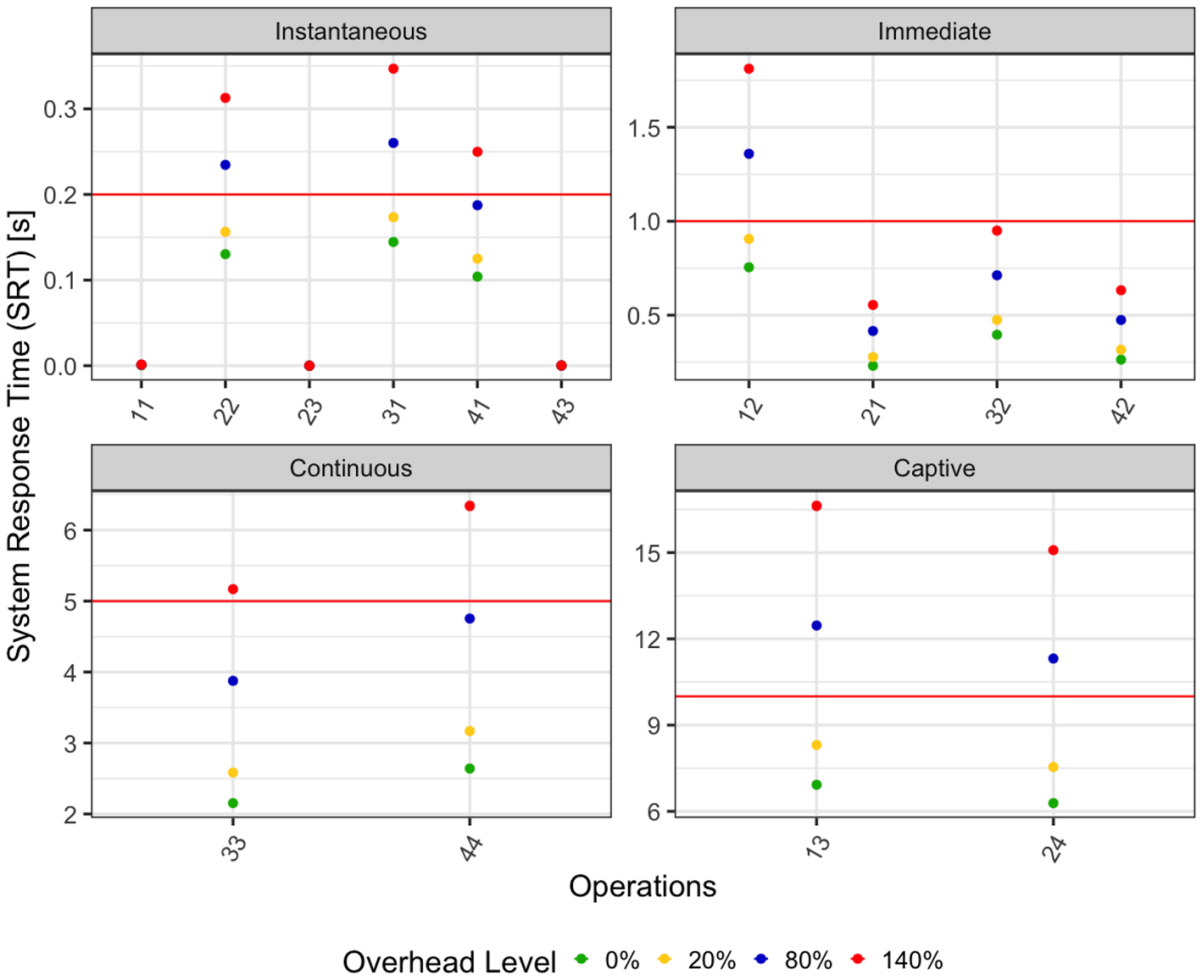
SRT when various overhead levels are applied. The horizontal red line shows the maximum expected time for the operations in that category

These operations have been exposed to three overhead values: 20%, 80%, 140%. Figure 3 shows the mean value of the resulting SRT for all the operations.

Depending on the resulting SRT, an operation can be *in time*, that is, its SRT is below the threshold for its category, or *slow*, that is, its SRT is above the threshold for its category. In the next sections, we analyze the impact of the overhead and how it is perceived by the users both considering the relative increment of the SRT and the total SRT distinguishing between in time and slow operations. We however exclude immediate operations from the analysis of the total SRT because too few operations are slow after the introduction of the overhead.

### Data analysis techniques

Even though at the family level there is a third level of clustering (i.e., experiment), the experiments’ intra-class correlation coefficient (ICC) was equal to 0 at family level, and therefore, results would not be affected by the higher level of clustering (i.e., subjects within experiments) (McNeish et al., 2017). Consequently, we move on to analyzing all the data from the three replications as a unique one (a family), without the need of keeping the replication as a factor. By using this analysis strategy, we can gain evidence out of a family of experiments, deriving stronger results than with the analysis of the individual experiments. All the answers collected during the experiments, and the R^2^ scripts used to analyze them, are available online at https://github.com/danielabriolaUnimib/SRToverheadExperiment.

**Table 4:** Distribution of participants within replicas

	**G1**	**G2**	**G3**	**G4**	**G5**	**G6**	**G7**	**G8**	**Total**
1st replica	6	6	6	6	6	6	6	6	48
2nd replica	3	3	3	2	2	2	2	2	19
3rd replica	4	4	4	4	4	4	4	4	32
**Total**	13	13	13	12	12	12	12	12	99

The experiment has been replicated three times: the first replication involved 48 participants, the second 19, and the third 32. The three replicas were performed at the same institution, in the same laboratory, and following exactly the same procedure. Besides, in Table 4, we show how participants were distributed in the different experimental groups within the different replicas.

We analyzed the data of the group of replications with generalized estimating equations (GEEs) (Ghisletta & Spini, 2004). GEEs are parametric statistical models traditionally used in biology and epidemiology to analyze longitudinal and clustered data (such as patients over time, or patients within hospitals). GEEs can be seen as traditional multiple regression models where parameter estimates and standard errors are corrected taking into account the clustering structure of the data (McNeish et al., 2017).

We chose parametric statistical models (such as GEEs), rather than non-parametric statistical models (such as the Wilcoxon test (Field, 2013)) because we are interested in analyzing the effect of the interaction of various factors (i.e., operation category, overhead level, and operation category order) on results—rather than the effect of single factors as in traditionally used non-parametric models (Field, 2013).

We relied on GEEs, rather than on other typically used parametric statistical models for analyzing longitudinal data, such as repeated-measures ANOVA because GEEs allow assessing the effect of various variance-covariance matrices (e.g., such as exchangeable, autoregressive, unstructured (Wang, 2014)) on results. In other words, GEEs allow assessing the effect of assuming different types of relationships across the participants’s scores over time. This allows assessing the robustness of the results to different specifications and, thus, increasing the reliability of the results.

Finally, we preferred GEEs over linear mixed models (LMMs (Verbeke, 1997)) because GEEs provide accurate results despite misspecifications in the variance-covariance matrix (contrary to LMMs (McNeish et al., 2017; Zorn, 2001)) and also because GEEs make fewer distributional assumptions than LMMs (e.g., the normality assumption of the random effects in LMMs (McNeish et al., 2017)), that eases the interpretation of results (e.g., in case data transformations were necessary with LMMs). For an introductory text on GEEs and their main advantages and characteristics, we refer to McNeish et al. (2017).

We fitted a GEE with the following factors: overhead levels, operation category order, and operation category. In particular, we fitted a GEE with the main effects of such factors, their two-way and three-way interactions. We included all the interactions among the factors because a priori any of them may alter the values of the others. In other words, there is no current evidence in SE to suggest that the factors are independent as it may be considered if we did not include the interactions among them^3^.

GEEs are built on a series of assumptions (McNeish et al., 2017):

1. All relevant predictors are included. This assumption is satisfied as we want to assess the three-way interaction between overhead levels, operation category order, and operation category on a Likert scale from 1 to 5 dependent variable.^4^
2. Participants are not clustered within a larger unit, or the clustering at the higher level is negligible. This assumption is satisfied because the intra-class correlation coefficient at the experiment level (i.e., the higher level) equals to 0.
3. A sufficient sample size shall be available at the cluster level. A total of 99 subjects participated in the three replications. At least ten subjects are needed for GEE’s estimates to be reliable (McNeish et al., 2017). Therefore, this assumption is satisfied.
4. A reasonably close covariance matrix shall be selected to represent the correlation among cluster-level (i.e., subject) scores. A total of 14 measurements are taken per subject (one measurement per each operation within the tasks, operations that belong to a specific operation category). An exchangeable and an unstructured covariance matrix were selected to analyze the data. In view of the similarity of results, we opted to analyze the data with the most parsimonious covariance matrix (i.e., exchangeable structure).

We judge the statistical significance of the findings based on *p*-values. Particularly, we judge findings based on the magnitude and sign of the pairwise contrasts that we perform (i.e., the estimate column of Tables 6, 7 and 8). We only show the pairwise contrasts whose *p*-values are lower than 0.1 (and thus, show the largest effect sizes to focus on the main results).

We also repeated this analysis considering the *Total SRT* of an operation instead of the overhead level as a factor, to study the impact of the overhead from a complementary perspective. The total SRT of an operation can be either *in time*, if the response time of an operation is below its expected SRT, or *slow*, if the response time of an operation is higher than its expected SRT. The expected SRT is determined by the operation category.

The following two sections present the results obtained when considering the relative overhead level (Section 6.3) and the total SRT (Section 6.4) as a factor, respectively.

### Results considering the overhead level as a factor

A GEE model was fitted to analyze the family of experiments. An exchangeable matrix-covariance structure was fitted to analyze the data. According to the fitted model, the correlation among the experimental subjects is 0.152.

Table 5 shows the results of the ANOVA table corresponding to the GEE fitted to analyze the family.

**Table 5:** ANOVA results of the family

**Parameter**	**Degrees of freedom**	*p* **-value**
**Operation category**	3	<**0.001**
Overhead level	3	0.330
Operation categories order	1	0.122
Operation category*overhead level	9	**0.035**
Operation category*operation categories order	3	**0.005**
Overhead level*operation categories order	3	0.105
Operation category*overhead level*operation categories order	9	0.722

*significant interactions are highlighted in bold

As it can be seen in Table 5, the three-way interaction operation category*overhead level*operation categories order is not significant. Two two-way interactions operation category*operation categories order, operation category*overhead level as well as the factor *operation category* are significant with a *p*-value $< 0.05$.

The third two-way interaction overhead level*operation categories order and the factor *operation categories order* are not significant, since their *p*-values $> 0.05$.

After fitting the GEE, we provide a series of interaction plots and pairwise contrasts to answer our research questions (RQ1, RQ2, and RQ3). We assess the statistical significance of the findings based on the contrasts’ *p*-values (last column of Tables 6, 7 and 8). We assess findings based on the contrasts’ effect sizes (ES column of Tables 6, 7 and 8), which were estimated by computing the least-squares means (predicted marginal means) for the specified factors of the experiment.

**Table 6:** Statistical significant contrasts between operation categories order

**Grouping variables**		**Contrast information**			
**Operation category**	**Overhead level**	**Contrasts**	**Estimate**	**ES**	*p* **-value**
Continuous	0	Capt. Cont. - Cont. Capt.	0.500	0.226	0.023
	20	Capt. Cont. - Cont. Capt.	0.817	0.250	0.001
	80	Capt. Cont. - Cont. Capt.	0.542	0.270	0.045
Captive	20	Capt. Cont. - Cont. Capt.	0.417	0.250	0.096

Legend: *ES* effect size

Table 6 shows the effect size and *p*-value of the statistically significant contrasts for operation category order (captive - continuous vs. continuous - captive), grouped by overhead level and operation category.

Table 7 shows the effect size and *p*-value of the statistically significant contrasts between overhead levels, grouped by operation categories order and operation category.

**Table 7:** Statistical significant contrasts between overhead levels

**Grouping variables**		**Contrast information**			
**Operation category order**	**Operation category**	**Contrasts**	**Estimate**	**ES**	*p* **-value**
Captive - continuous	Immediate	0–140%	0.300	0.123	0.069
	Continuous	20–140%	0.708	0.257	0.029
	Captive	20–140%	0.708	0.278	0.053

Legend: *ES* effect size

**Table 8:** Statistical significant contrasts between operation categories

**Grouping variables**		**Contrast information**			
**Operation category order**	**Overhead**	**Contrasts**	**Estimate**	**ES**	*p* **-value**
Captive - continuous	0	Instantaneous - immediate	$-$0.140	0.044	0.007
		Instantaneous - captive	0.436	0.170	0.050
		Immediate - captive	0.577	0.166	0.003
		Continuous - captive	0.801	0.168	< 0.001
	20	Instantaneous - continuous	$-$0.625	0.139	< 0.001
		Immediate - continuous	$-$0.583	0.173	0.004
		Continuous - captive	0.708	0.216	0.006
	80	Instantaneous - captive	0.736	0.229	0.007
		Immediate - captive	0.708	0.218	0.006
		Continuous - captive	0.667	0.160	< 0.001
	140	Instantaneous - captive	0.681	0.253	0.036
		Immediate - captive	0.646	0.227	0.023
		Continuous - captive	0.708	0.216	0.006
Continuous - captive	20	Instantaneous - captive	0.500	0.183	0.032
		Immediate - captive	0.404	0.169	0.079
	80	Instantaneous - continuous	0.458	0.187	0.067
		Instantaneous - captive	0.792	0.272	0.019
		Immediate - continuous	0.417	0.154	0.035
		Immediate - captive	0.750	0.221	0.004
	140	Instantaneous - captive	1.237	0.225	< 0.001
		Immediate - captive	1.186	0.194	< 0.001
		Continuous - captive	0.708	0.171	< 0.001

Legend: *ES* effect size

Table 8 shows the effect size and *p*-value of the statistically significant contrasts between operation categories, grouped by operation categories order and overhead.

In Section 7, we plot the data, report statistically relevant interactions (i.e., pairwise contrasts whose *p*-values are lower than 0.1), and discuss the interpretation of these results.

### Results considering the total SRT as a factor

A GEE model was fitted to analyze the family of experiments. An exchangeable matrix-covariance structure was fitted to analyze the data. According to the model fitted, the correlation among the experimental subjects scores equals to 0.152.

Table 9 shows the results of the ANOVA table corresponding to the GEE fitted to analyze the family.

**Table 9:** anova results of the family considering the total SRT

**Parameter**	**Degrees of freedom**	*p* **-value**
**Operation category**	3	$\varvec{< 2 \times 10^{-16}}$
**Total SRT**	1	**0.0017**
Operation categories order	1	0.1244
Operation category*total SRT	3	**0.0129**
Operation category*operation categories order	3	**0.0018**
Total SRT*operation categories order	1	0.4322
Operation category*total SRT*operation categories order	3	0.1635

*Significant interactions are highlighted in bold

As it can be seen in Table 9, the three-way interaction operation category*Total SRT*operation categories Order is not significant. Two two-way interactions operation category*operation categories order, operation category*Total SRT, and the factors *operation category* and *Total SRT* are significant at the 0.05 level.

After fitting the GEE, we provide a series of interaction plots and pairwise contrasts to answer our research questions (RQ1, RQ2, and RQ3). We assess the statistical significance of the findings based on the contrasts’ *p*-values. We assess the findings based on the contrasts’ effect sizes, which were estimated by computing the least-squares means (predicted marginal means) for the specified factors of the experiment.

Table 10 shows the effect size and *p*-value of the statistically significant contrasts for operation categories order (captive - continuous vs. continuous - captive), grouped by total SRT and operation category.

**Table 10:** statistical significant contrasts between operation categories order

**Grouping variables**		**Contrast information**			
**Operation category**	**Total SRT**	**Contrasts**	**Estimate**	**ES**	*p* **-value**
Continuous	0	Capt. Cont. - Cont. Capt.	0.5	0.226	0.023

Legend: *ES* effect size

Table 11 shows the effect size and *p*-value of the statistically significant contrasts between total SRT, grouped by operation categories order and operation category.

**Table 11:** Statistical significant contrasts between *slow* and *in time* operations

**Grouping variables**		**Contrast information**			
**Operation category order**	**Operation category**	**Contrasts**	**Estimate**	**ES**	*p* **-value**
Continuous - captive	Captive	In time - slow	0.471	0.2067	0.0230
Captive - continuous	Captive	In time - slow	0.377	0.1851	0.0420

Legend: *ES* effect size

Table 12 shows the effect size and *p*-value of the statistically significant contrasts between operation categories, grouped by operation categories order and total SRT.

**Table 12:** Statistical significant contrasts between operation categories

**Grouping variables**		**Contrast information**			
**Operation category order**	**Total SRT**	**Contrasts**	**Estimate**	**ES**	*p* **-value**
Captive - continuous	In time	Instantaneous - continuous	−0.307	0.1102	0.0280
		Continuous - captive	0.620	0.1390	< 0.0001
	Slow	Instantaneous - captive	0.617	0.1874	0.0050
		Continuous - captive	0.671	0.2000	0.004
Continuous - captive	In time	Instantaneous - captive	0.522	0.1525	0.003
	Slow	Instantaneous - captive	0.938	0.1830	< 0.0001
		Continuous - captive	0.523	0.2010	0.0460

Legend: *ES* effect size

## Findings

This section discusses the findings related to the three research questions introduced in Section 3 based on the results reported in Section 6. For each research question, we first discuss the results obtained considering the overhead level factor, then we discuss the results for the Total SRT, and we conclude by answering the research question.

### RQ1: to what extent do different overhead levels impact developers’ perceptions?

#### Overhead levels

**Fig. 4:**
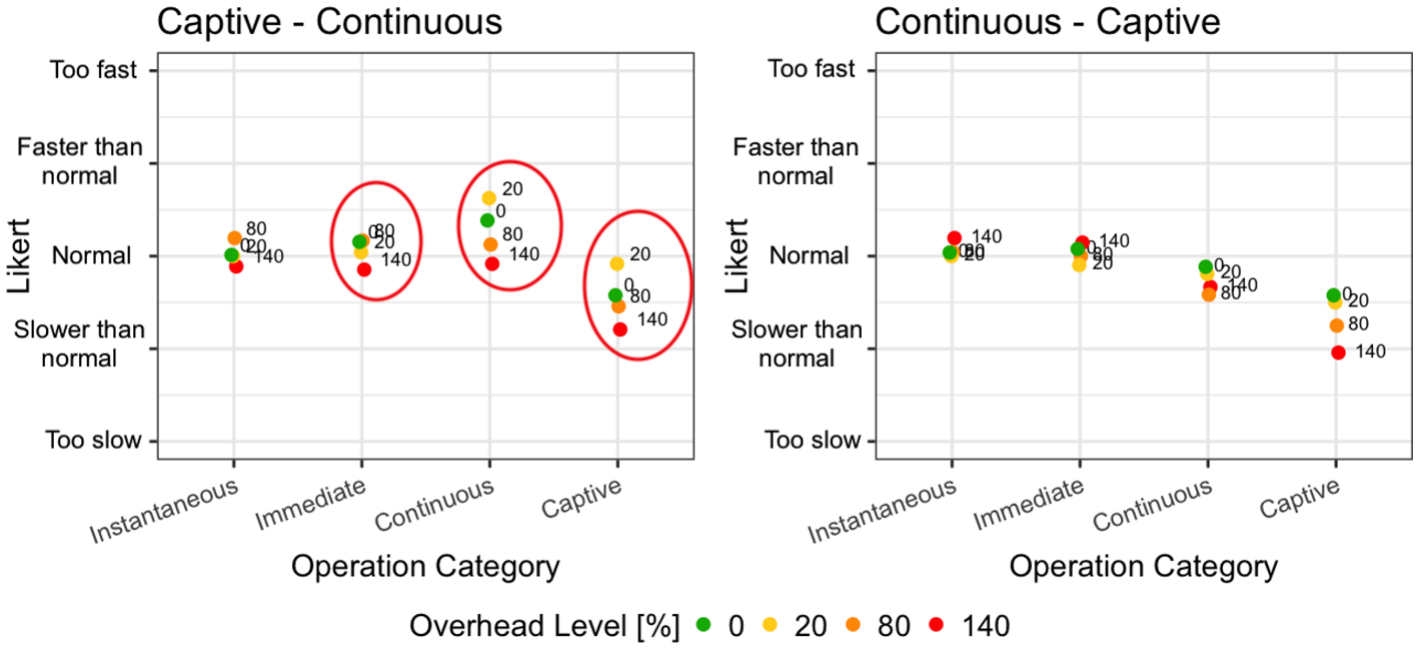
Interaction plot divided by operation categories order and operation category, and colored by overhead level

Figure 4 shows how the overhead levels have been perceived by the subjects depending on the order of execution of the operation category order (captive - continuous vs continuous - captive) and depending on the operation category that is performed (instantaneous, immediate, continuous, captive). The circles capture groups with statistically significant differences according to the analysis presented in Table 7, where immediate, continuous, and captive operations are perceived differently for overhead values equal to 20% and 140%.

In all the cases, an overhead level until 80% had a negligible impact based on the feedback provided by the subjects involved in the study for immediate, instantaneous, and continuous operations. The fact that users can tolerate a non-trivial overhead is extremely interesting since it suggests that the field can feasibly accommodate analysis procedures running concurrently with applications, even when applications are actually interactive.

Overhead levels up to 140% have been in some cases distinguished by the users compared to lower overhead levels. This indicates that overhead levels between 80 and 140% represent boundary values and higher overhead levels may impact the user experience in a significant way.

It is important to remark that this result has been obtained in the context of overhead levels introduced for a limited number of operations, between 3 and 4 in our study, and that we cannot conclude anything about overhead levels persisting for a higher number of operations, such as dozens of operations.

#### Total SRT

**Fig. 5:**
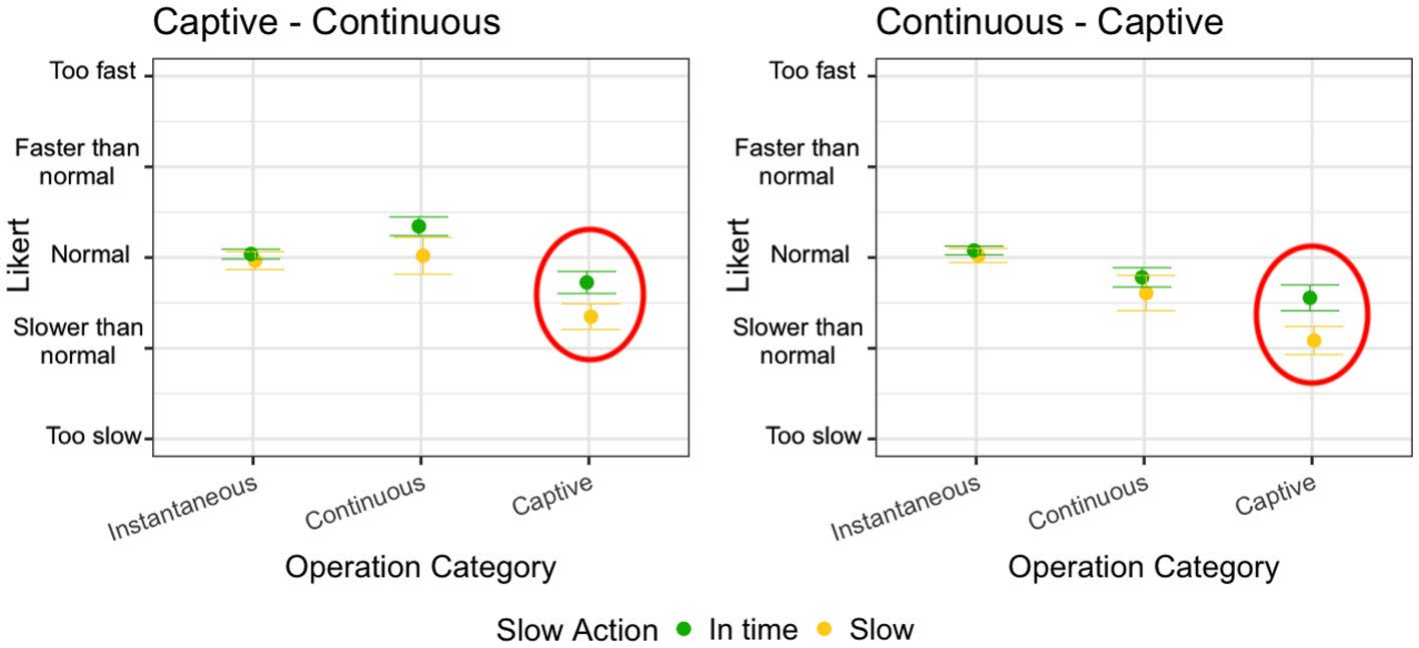
Perceived SRT with respect to a *slow* or *in time* total SRT

Figure 5 shows the box plot of the perceived SRT for the various categories of operations, distinguishing the case the operation is *in time* or *slow*. We do not report results for immediate operations since they are seldom *slow* in our experiment, as discussed in Section 6.1. The red circles highlight the statistically significant difference as reported in Table 11.

The result is quite clear. *Slow* actions are mostly not recognized as such by the participants to the experiment with the exception of captive operations, which are the operations with the longest duration. This confirms the intuition that non-trivial overhead levels can be tolerated by users of interactive applications, as long as the executed operation is not a long-lasting operation.

#### Answer to RQ1

The two perspectives of the analysis show that the participants can hardly perceive overhead levels below 80%, especially for operations that are not captive. Significantly increasing the duration of captive operations (e.g., increasing the duration by more than 80% reaching a total execution time beyond 10 s, so turning it into a *slow* operation) should be done carefully because it has a non-negligible probability to annoy users.

### RQ2: is the perception of the overhead level dependent on the operation category?

#### Overhead levels

**Fig. 6:**
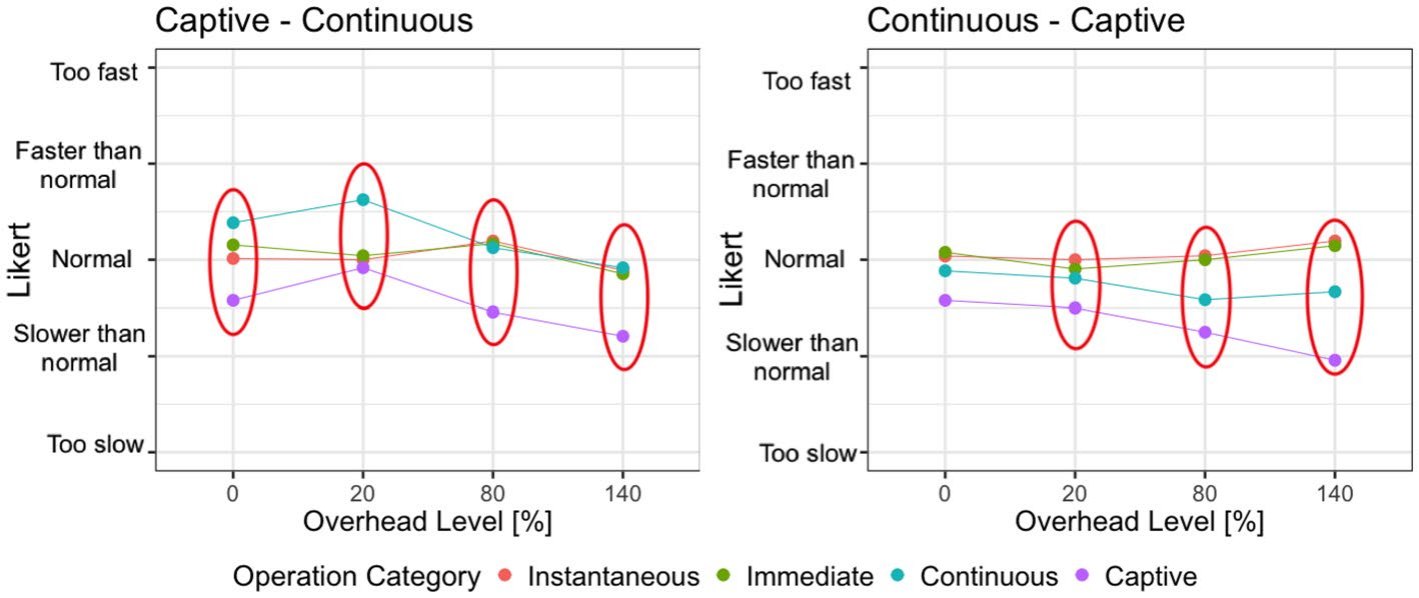
Interaction plot divided by operation categories order and overhead level, and colored by operation categories

Figure 6 shows how the operation category impacted on the perception of the overhead, depending on the overhead level (0%, 20%, 80%, 140%) and the operation category order (continuous - captive and captive - continuous). The circles capture statistically significant differences according to the analysis presented in Table 8.

We reported significant differences in all the groups and for all the cases, but mainly the differences involve either a captive or a continuous operation and an operation in another category. The plot clearly shows that the overhead introduced in captive and continuous operations is systematically perceived as worse than the overhead introduced in immediate and instantaneous operations. On the contrary, little differences are reported between simple operations (instantaneous and immediate). This suggests that techniques working in the field should consider the nature of the operation that is executed to control their impact on the perceived system response time.

**Fig. 7:**
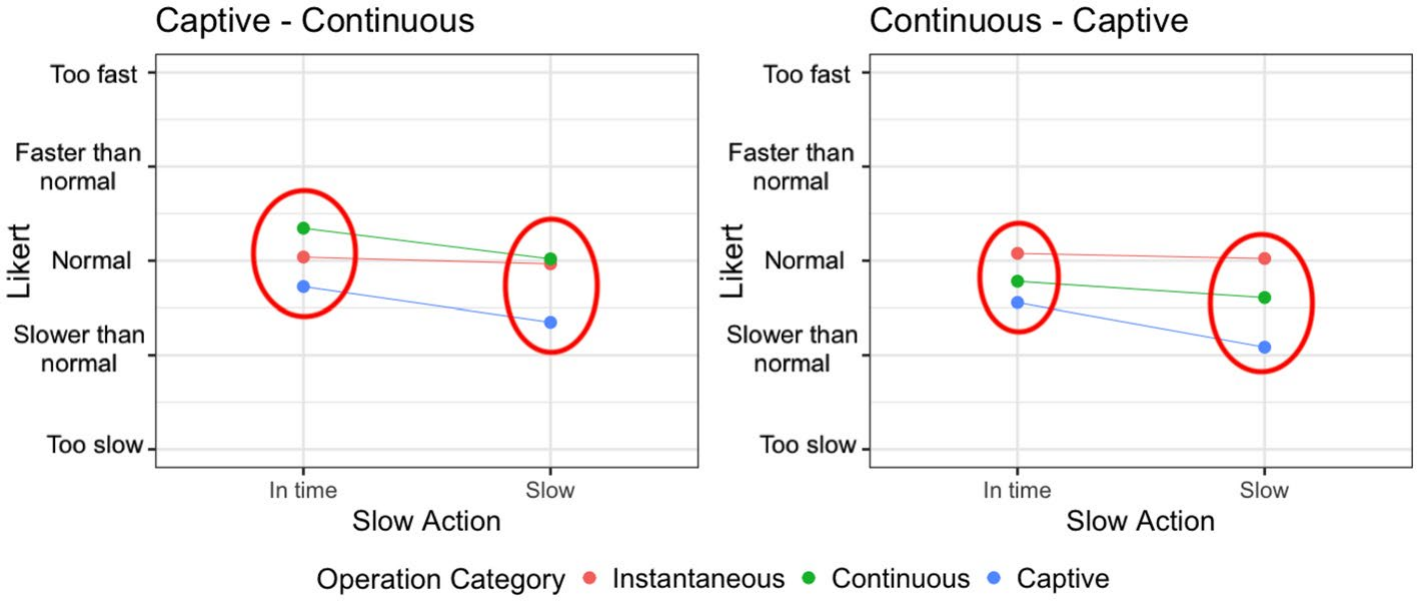
Perceived SRT considering the total SRT of the operations

#### Total SRT

Figure 7 shows how the operation category impacted the perception of the SRT depending on the total SRT of the operations (*in time* or *slow*) and the operation categories order (continuous - captive and captive - continuous).

The figure shows that the overhead does not impact all the operations equally. In particular, captive operations are impacted more heavily (the slope of the line is higher) compared to continuous and instantaneous operations. Indeed, this results in statistically significant differences between captive operations and the rest of the operations, as reported in Table 12. Finally, continuous operations also show a different trend than the simplest operations, overall confirming that operations that last longer are perceived more negatively than quick operations.

#### Answer to RQ2

The analysis of RQ2 shows that users better tolerate the overhead introduced in simple operations compared to the overhead introduced in complex operations, with captive operations being the ones more sensitive to overhead.

### RQ3: does the order of execution of complex operations affect the developer’s perception of SRT?

#### Overhead levels

**Fig. 8:**
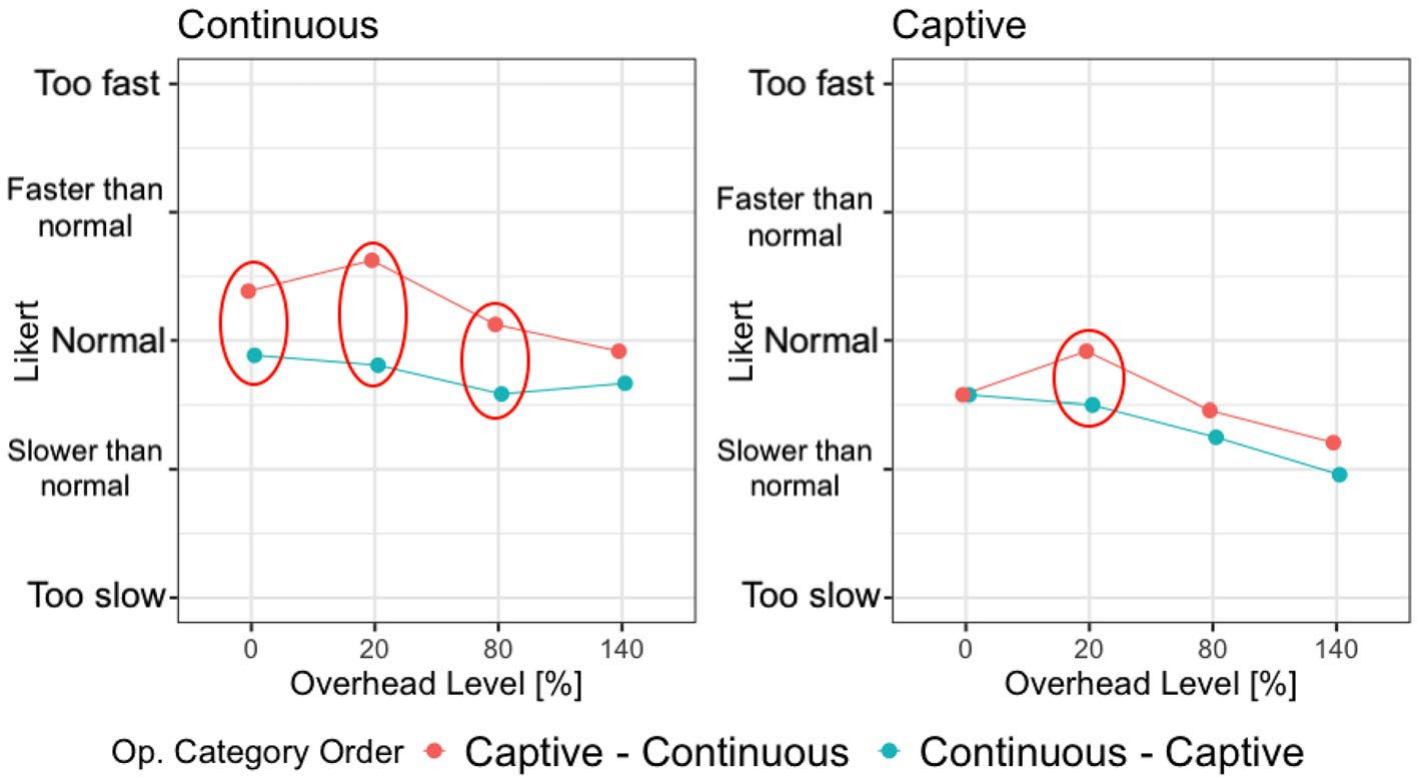
Interaction plot divided by operation category and overhead levels, colored by operation categories order

Figure 8 shows how the operation categories order (captive - continuous vs continuous - captive) impacts on the perception of the SRT of continuous and captive operations, depending on the overhead level (0%, 20%, 80%, 140%). The circles capture statistically significant differences according to the analysis presented in Table 6.

The most significant results have been obtained for continuous operations. In fact, the same operations exposed to the same overhead levels are perceived in a radically different way if executed before or after captive operations. A continuous operation is perceived better when executed after a captive operation, which takes longer than the continuous operation, and may potentially ease the acceptance of the time required by the continuous one. We hypothesize that the execution of a captive operation, which may require multiple seconds to complete, puts the user in a rather negative mood about the response time of the system and this affects the evaluation of the following operations. On the contrary, when the continuous operation is executed without the bias of the captive operation, its response time is perceived as worse. Finally, note that when the overhead is as high as 140%, the response time is too long in both cases and there is no significant difference in the responses of the users (they dislike both). This is consistent with the results for RQ1 that show that when the overhead increases above 80%, its effect can be recognized by users.

Regarding captive operations, they are long-lasting operations whose perception of the SRT does not change much whether they are executed before or after a continuous operation, although it is consistently worse to execute the captive operation (i.e., a long-lasting operation) after the continuous operation (i.e., a reasonably fast operation) making the user perceive the long-running operation even worse than it is. A significant difference is however present only for overhead equals to 20%. With no overhead or higher overhead levels, the response time is perceived as slow regardless of its context.

#### Total SRT

**Fig. 9:**
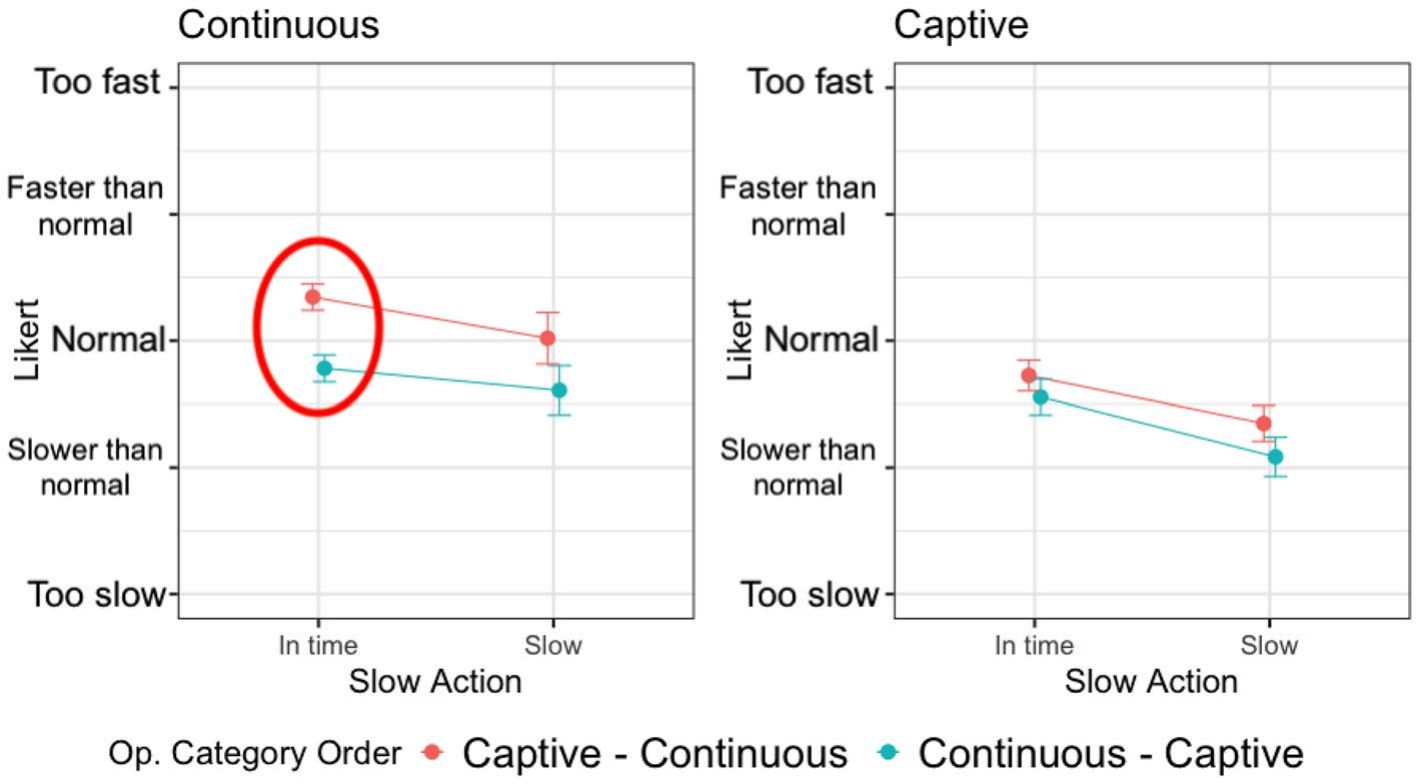
Interaction plot divided by operation category and *slow or in time* operations, colored by operation categories order

Figure 9 shows how the SRT is perceived by the subjects when the complex operations are *in time* or *slow*, considering their order of occurrence. Results are coherent with the analysis based on the overhead levels. The order of execution of the operations impacts on continuous operations only. When the SRT increases, both because of the overhead (see the case of *slow* continuous operations and *slow* captive operations) and because of the nature of the operation (see the case of *in time* captive operations), the order does not produce significant differences.

The significant difference is in the continuous operations that are perceived faster when executed after the captive operation, that is, the captive operation sets up a bad expectation about the SRT of the application that makes the subjects perceive positively the SRT of the following continuous operation (since it is faster than they expected).

#### Answer to RQ3

Based on the reported results, the perception of the overhead can be affected by the execution of captive operations, especially for the following continuous operations, which might be perceived as executing faster than they are. On the contrary, captive operations are not significantly influenced by their context of execution.

## Study with professional developers

### Design and setup of the experiment

The results obtained with computer science students may not generalize to professionals. We thus designed an additional small-scale study involving 12 professionals aimed at validating the obtained findings. We recruited professionals on a volunteer basis from our contacts working in companies in Milan and Luxembourg.

Since involving professionals in a fully controlled in-lab experiment, as done with students, is practically infeasible due to the strong time and organizational constraints professionals are subject to, we designed a shorter online study targeting the key observations reported in the answers to RQs1–3. Still, the online study has the same structure of the study with students.

The key findings obtained with students that we want to validate are the following ones:

- RQ1: Developers can hardly perceive overhead levels below 80%, especially for captive operations, while increasing the overhead over 80% for captive operations may easily annoy them.
- RQ2: Developers better tolerate the overhead introduced in simple operations compared to the overhead introduced in complex operations, with captive operations being the ones more sensitive to overhead.
- RQ3: Continuous operations following captive operations might be perceived as executing faster than they are, while captive operations are not significantly influenced by the context of execution.

To be as similar as possible to the original experiment, while making sure to expose the professionals to the right overhead, we used recorded videos of the tasks. In particular, we recorded the executions of the exact four tasks presented in Section 5.2, using GUI interactions rather than keyboard interactions to let professionals clearly perceive when each interaction starts, and also making sure each recorded action matches with the expected category.

We then edited the videos to introduce the required overhead: we only used two overhead levels (20% and 140%), since they are sufficient to validate the key findings listed above. We preserved the category order as a factor for the experiment, thus exposing subjects to the two tasks ending with captive operations followed by the two tasks ending with continuous operations, or vice versa.

In a nutshell, to validate the main findings, the design still has to cover two overhead levels, four operation categories, and two operation categories orders, as summarized in Table 13.

**Table 13:** Factors and treatment levels for the validation experiment

**Factors**	**Treatment levels**
Overhead level	20%, 140%
Operation category	Instantaneous, immediate, continuous, captive
Operation categories order	Continuous - captive, captive - continuous

**Table 14:** Design for the validation experiment

**Tasks**	**Operation category order**	**Applied overhead level**	
		**20%**	**140%**
T3 T4 T1 T2	Continuous - captive	G1	G3
T1 T2 T3 T4	Captive - continuous	G2	G4

We consistently used the same design, *between overhead levels*, for both the students and the professionals. All the professionals have been homogeneously distributed across four groups G1–G4 (see Table 14). Groups G1 (20% overhead) and G3 (140% overhead) first experienced the two tasks terminating with a continuous operation and then the two tasks terminating with a captive operation, while groups G2 (20% overhead) and G4 (140% overhead) experienced the opposite order.

When doing the experiment, each subject used a link to access an online form, where he/she can download the instructions, the videos, and the guide to perform the activity. We used a different link for each group, to prevent any risk a subject may perform the tasks in the wrong order. Professionals submitted their assessment through an online form. We include all the documents and artifacts needed for this study in the material shared online at https://github.com/danielabriolaUnimib/SRToverheadExperiment.

### Results

**Fig. 10:**
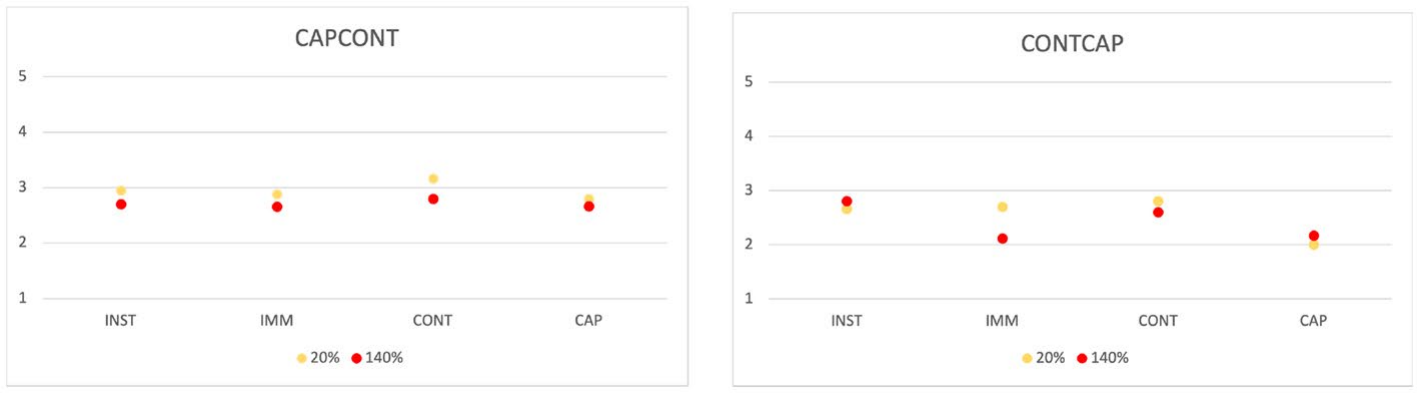
Interaction plot divided by operation categories order and operation category and colored by overhead level. See Table 15 for the *Y* axis number conversion. The value of the y axis indicates how the operation is perceived (5=too fast, 4=faster than normal, 3=normal, 2=slower than normal, 1=too slow)

We first discuss the results for RQ1. Figure 10 shows the interaction plot divided by operation categories order and operation category and colored by overhead level. Professionals confirmed that small overhead levels (e.g., overhead equals to 20%) can be hardly perceived (values close to normal in seven cases out of eight). They also confirm that higher overhead levels can often be tolerated (overhead levels equal to 140% are perceived as normal in six cases out of eight). Also, in this case, high overhead applied to captive operations may annoy the subjects, but this phenomenon is context-dependent, that is, captive operations are perceived slower if executed after continuous operations. The context is particularly relevant in this case, because an overhead of 20% has been perceived as annoying for captive operations again only when executed after continuous operations. In a nutshell, professionals confirm the findings for RQ1, while reporting a stronger dependence on the context for captive operations.

**Table 15:** *Y* axis number conversion for Fig 10.

**How the operation is perceived**	**Value**
Too fast	5
Faster than normal	4
Normal	3
Slower than normal	2
Too slow	1

The results for RQ2 show that simple actions are less sensitive to overhead than complex actions, captive operations in particular. This is also confirmed by professionals, with captive being the only type of operation assessed as too slow for both an overhead of 20% and 140% (see the right plot in Fig. 10). Again, contrarily to students, this result is context-dependent, since we have not observed the same for captive operations executed before continuous operations.

The results for RQ3 show that continuous operations are perceived faster when executed after captive operations. Professionals report a result that is conceptually similar, although practically different. That is, they perceived captive operations executed after continuous operations slower than they are. This means that the order of the operations tends to influence professionals negatively (captive operations perceived slower when executed after continuous ones) rather than positively (continuous operations perceived faster when executed after captive operations), as observed for students.

In a nutshell, professionals confirm the results obtained with students with two caveats: (1) the operation order plays a bigger role on perception, and (2) the operation order tends to bias the perception negatively rather the positively. We may explain these differences as the likely stronger expectations that professionals have on the performance of the executed operations, thus being slightly more sensitive to overhead, especially for specific orders of operations.

## Threats to validity

In this section, we discuss the threats to the validity of our study.

The main threat to *conclusion validity* concerns the possible lack of causality for the significant relationships that have been identified. To mitigate this risk, we considered the strength of the reported evidence in the discussion of the results. Moreover, to mitigate the risk that an interference is produced by the organization in groups of the participants, we assigned people to groups randomly.

The main threats to *construct validity* concern the way the overhead has been introduced in the software and the clarity of the tasks performed by the subjects. We controlled the overhead introduced in the software by carefully designing the aspects based on the performance of the machines in the lab used for the experiment. Note that all the machines have exactly the same hardware and software. We tested the consistency of the behavior of our software on every machine of the lab.

Some participants might be relatively confident with experimented applications and tasks. To mitigate this threat, we asked the participants to indicate if the activity they performed was clear in the exit questionnaire: 96.3% of the answers were positive, and only 3.7% of the participants found some unclear aspects. We can thus assume that the values collected for the response variable were sufficiently accurate.

The *internal validity* threats have been addressed in Section 4 with the careful discussion of the design decisions that motivated our final design.

The main threat to the *external validity* is the representativeness of the participants and the application we used. The computer science students we recruited acted as regular computer programmers, so their student status mitigates any threat on the external validity of the experiment. To further mitigate this threat, we designed a small-scale study involving 12 professional developers to assess the key findings obtained with students. On the other hand, it is known that the perception of time can be different for young people, adults, and elderly people (Duncan et al., 2005). Our study is thus representative of the perception of young people.

We studied the perceived SRT for interactive desktop applications considering the case of IDE tools, Eclipse in particular, while covering multiple categories of operations. While the results are informative on how the overhead is perceived for this kind of computer application by developers, they are not guaranteed to hold for different applications, different interaction sequences and above all for Web applications, where the network may play a relevant role on the SRT, and mobile applications, where the different hardware and interaction modalities may have an impact. Additional studies are necessary to extend our findings to these other domains.

## Actionable results

In this section, we summarize the main actionable findings of our study and discuss their impact on analysis strategies working in the field within development tools. To give concrete evidence of their actionability, we also exemplify how they can be used to improve two monitoring techniques designed for the field.

### Main actionable findings

#### F1: monitoring and analysis routines can consume non-trivial resources for a limited number of interactions

Developers seldom perceived the variations in the SRT even for the largest overhead levels. This implies that *applications can be safely slowed down to support field analysis and monitoring*, at least for sequences of limited length (about four operations). This result opens to the design of families of monitoring and analysis solutions that can *opportunistically consume significant resources* for a limited amount of time.

#### F2: monitoring and analysis routines should control the overhead introduced in long-lasting operations

Developers tolerated well the overhead introduced in simple operations (instantaneous and immediate) and also in continuous operations but revealed to be quite sensitive to the overhead introduced in captive operations, especially if executed after continuous operations. This introduces a significant risk of annoying the developers if the operation that is slowed down requires between 5 and 10 s to complete. As a consequence, monitoring and analysis solutions working in the field could be more effective if activated *selectively*, *avoiding to overlap with captive* operations as much as possible.

#### F3: monitoring and analysis routines should be aware of the execution context

Based on our study, the recent history of execution influences the perceived SRT. In particular, we studied this phenomenon for continuous and captive operations, and we discovered that continuous operations are perceived faster by students if executed after captive operations, and dually captive operations are perceived slower if executed after continuous operations. This introduces the challenge of having monitoring and analysis strategies that *keep track of the recent history* of the execution and exploit the context to *opportunistically increase or reduce their activity*.

### Improving monitoring strategies: two examples

The reported findings can be exploited to improve field monitoring techniques, for example, controlled burst recording (CBR) (Cornejo et al., 2017b, 2020b) and delayed saving (Cornejo et al., 2019).

CBR (Cornejo et al., 2017b, 2020b) is a monitoring technique that can record bursts (sequences) of function calls whose activation and deactivation are controlled by the operations performed by the users on the target application: when a new user operation is started (e.g., the user clicks on a button), the monitor records, with a given probability, every function call that is executed until the application returns a feedback to the user. CBR also records information about the state of the application before and after the burst is collected, to finally obtain a finite state model that represents how the monitored application is used by its users, adding no more than 125% of overhead on average.

The results obtained with the study described in this paper can be used to improve CBR in at least two ways. First, since users can hardly perceive an overhead up to 140% when introduced for a limited number of interactions (findings F1 and F2), bursts could be now obtained from multiple (four from this paper) operations performed sequentially instead of being limited, as in the current version, to record the execution of only one single operation with a given probability.

Second, CBR can be extended to be aware of the nature of the operation that is executed (finding F2) and of the context (finding F3) to adjust the sampling rate and the data recording behavior with respect to it.

The discussed set of improvements can lead to more accurate and complete traces extracted faster, improving the overall capability of the monitoring technique to discover up-to-date information about the behavior of developers.

Delayed saving (Cornejo et al., 2019) is a technique that allows to save extensive amount of data in memory while waiting for computer inactivity to save the data to disk without annoying users. To inexpensively keep data in memory while waiting to be saved, delayed saving does not copy values but maintains reference to live objects. As a consequence, some variables may change their values by the time they are saved to disk, introducing data inconsistencies. Experiments with the Eclipse IDE show that delayed saving can limit the overhead to about 5% while saving 85% accurate data.

The results in this paper show that higher overhead levels can be definitely introduced, at least sporadically, without impacting on developers (findings F1 and F2). This suggests that the percentage of inaccurate data could be reduced by saving data to files for longer time, not only during computer inactivity but also partially overlapping with the user activity, trading a slightly higher overhead for more accurate data. Again, the saving strategy could be also sophisticated taking the nature of the operation that is performed into account (finding F3).

## Related work

In this section, we frame our work in the context of related areas. In particular, we discuss how it relates to studies about the perception of the system response time, about field monitoring and analysis, and more specifically about monitoring developers while working in their environment.

### Perception of SRT

There are multiple studies related to the perception of the SRT in the context of the research in HCI and psychology. For instance, the perception of time is a complex subject of study in psychology, and several researchers investigated it. Relevant to our experiment, Duncan et al. (2005) studied how the perception of time changes with age discovering that young people, adults and elderly people perceive time differently. This is why we selected subjects of similar age. Our study is thus representative of how young developers perceive the overhead.

In the HCI area, there are a number of studies about the satisfaction of the user concerning the SRT of an application. For example, Ceaparu et al. (2004) studied the interactions with a personal computer that causes frustration. In their experiment, users were asked to freely work at home for at least an hour on a computer and then to compile a questionnaire about what was frustrating. Although this experiment is different in both the design and the aim from ours, the results show that SRT delays (e.g., a too slow Web browser) might be the cause of a bad user experience. Some studies stressed the tolerance of the users in specific settings. For example, Nah (2004) investigated how long users are willing to wait for a Web page to be downloaded. Results show that users start noticing the slowdowns after 2-s delays and that do not tolerate a slow down of more than 15 s. A threshold of 15 s has been reported as the maximum that can be tolerated before perceiving an interruption in a conversation with an application also in other studies (Nielsen, 1999; Miller, 1968). Hoxmeier and Cesare (2000) studied how users react to overhead introduced in the transitions between Web pages and discovered that users can tolerate up to a 12-s delay. Yet other studies considered the impact of response delays and latency in crowd-powered conversational systems (CPCS)  (Abbas et al., 2022), mobile searches (Arapakis et al., 2021), and conversational agents (Funk et al., 2020).

While these findings are interesting, they focus on complementary aspects compared to our experiment. In fact, we are not interested in identifying the maximum overhead that a user can tolerate, but we are interested in the overhead that users cannot even recognize. In other words, we are not interested in stressing up to the limit the users, but rather to seamlessly introduce analysis and monitoring routines in software applications. Moreover, our study covers different classes of operations, including operations to move across windows and menus and also the execution of domain functionalities, instead of focusing on specific operations (e.g., transitions between Web pages). Finally, our experiment considers the specific case of developers working on their IDE.

Some studies demonstrated that perception might be affected by psychological factors, such as motivation and interest (Luo et al., 2017). Also, the animations visualized during the waiting time might have an impact on the perception of time (Söderström et al., 2018). Extending the study considering these factors is part of our future work.

### Field monitoring and analysis

Field monitoring and analysis solutions can significantly improve verification and validation methods since they can work with production data and can exploit the knowledge of the real interaction patterns between users and applications. In particular, field monitoring has been used to collect data that can support the reproduction and analysis of failures (Jin & Orso, 2012; Clause & Orso, 2007; Liblit et al., 2003; Jin et al., 2010), but also to collect additional evidence about the correctness of software systems, such as residual coverage data (Ohmann et al., 2016; Pavlopoulou & Young, 1999). Field data can also be exploited to validate software in-house according to patterns consistent with the usage scenarios observed in the field (Gazzola et al., 2023; Elbaum & Diep, 2005). Finally, data about correctness can be also collected proactively, by running tests in the field, rather than only observing the software passively (Bertolino et al., 2022; Ceccato et al., 2020; Hosek & Cadar, 2015).

Although these solutions can be beneficial for the quality of the software, they may degrade the performance of the applications and consequently affect the quality of the user experience if not carefully designed. So far the research focused on the design of mechanisms that can work in the field, possibly limiting their intrusiveness, but with limited understanding of the possible reaction of the users to the experienced overhead.

Some monitoring and data collection techniques have been specifically designed to limit the overhead introduced in the target application. Existing strategies include distributing the monitoring activity among the many instances of a same application (Bowring et al., 2002; Orso et al., 2002), collecting events probabilistically (Liblit et al., 2003; Jin et al., 2010; Bartocci et al., 2012), and collecting subsequences of events with ad-hoc strategies (Hirzel & Chilimbi, 2001; Cornejo et al., 2017b). While these approaches may reduce the overhead, how and if the overhead is perceived by users is not investigated in these papers.

### Monitoring developers

Improving the development process, and consequently, the productivity of developers and development teams, is indeed an objective of many software engineering practices. Improving the process normally requires collecting information about the behavior of the participants to the process, so that appropriate actions can be taken. Monitoring solutions targeting software developers can collect data of different types and at different granularity levels. For instance, the collected information might be about the development process (van der Aalst, 2012; Rubin et al., 2014) or about the individual operations performed by the developers while completing technical tasks (Meyer, Barton et al., 2017; Meyer, Murphy et al., 2017; Züger et al., 2017). The collected data can finally be used to take corrective actions about practices (Meyer, Barton et al., 2017), tools (Meyer, Murphy et al., 2017), and collaboration mechanisms (van der Aalst, 2012). Interestingly, a recent stream of work investigated the collection of behavioral data jointly with bio-metric data to support a number of software engineering tasks (Laudato et al., 2023; Couceiro et al., 2019).

In all these cases, suitable monitoring mechanisms must be in place to collect accurate data that can support decisions. However, there is also a need to collecting data transparently without annoying developers, possibly with developers not even noticing any slowdown in their systems. The study presented in this paper offers a first quantification of the amount of overhead that can be introduced into development tools without developers noticing the increased SRT. These results can be used to optimize the data collection strategies, as preliminary discussed in this paper.

## Conclusions

Development and operation are nowadays tightly integrated phases that are sometimes hard to distinguish one from the other. The operational environment is not only designed to provide services to users but is also conceived as a monitoring, analysis, and testing platform that can produce useful data to improve produces and processes. In particular, monitoring solutions can be used to obtain data directly from the workstations used by developers to optimize development practices, enhance software tools, and improve collaboration between developers.

So far, the research focused on how to enrich the operational environment paying limited attention on how the generated overhead may impact on the perception of SRT. In this paper, we presented an empirical study about how developers perceive delays introduced in their IDEs, distilling findings useful to design and tune solutions that operate in the field. For instance, our results indicate the levels of overhead that can be transparently introduced into the target application and the factors that should be controlled to make monitoring non-intrusive, such as the nature of the operation that is executed and its context of execution.

Even if the presented results primarily target developers, they represent a starting point for studying a broader set of situations where users of software applications have an understanding of the response time expected for the application they are using. Investigating the potential validity of our results in other domains is part of our future work. An additional direction of our future work consists of studying both how developers react to an overhead that persists for a long time and how developers react to an overhead introduced intermittently (e.g., the system is slowed down for a while, then it goes back to normal operation, then it is slowed down again, and so on). Results will allow us to elaborate an initial set of requirements and recommendations about how analysis and monitoring solutions should work in the field.

## Data Availability

The authors declare that the data supporting the findings of this study (including the anonymous answers of the participants, the questionnaires, the instructions, and the scripts for analyzing the data) are available online at https://github.com/danielabriolaUnimib/SRToverheadExperiment.

## Footnotes

1 SikuliX homepage http://sikulix.com/

2 https://www.r-project.org/

3 Some authors suggest building statistical models following either top-down or bottom-up strategies (West et al., 2014). In short, factors (and their interactions) in such approaches are either added or dropped based on their statistical significance levels (e.g., if the interaction is not statistically significant, then drop it). However, such strategies have been lately discouraged in mature experimental disciplines such as biology due to their heightened risk of committing Type I errors (Colegrave & Ruxton, 2017). Besides, as *p*-values are dependent upon the sample size (Cohen, 2016; Cumming, 2013), relevant factors (or interactions) may be dropped due to the presence of a small sample and not because they are not relevant in terms of effect size.

4 In this work, we consider the Likert scale as continuous. This approach has been commonly used in other disciplines (Norman, 2010; Wadgave & Khairnar, 2016).
